# Steric Hindrance of Glyphosate Adsorption to Metal (Hydr)oxides: A Novel Model Approach for Organic Matter-Mineral Interactions

**DOI:** 10.1021/acs.est.5c04207

**Published:** 2025-07-07

**Authors:** Bram Geysels, Jan E. Groenenberg, Tjisse Hiemstra, Héctor S. Apreza Arrieta, Arnoldus W. P. Vermeer, Rob N. J. Comans

**Affiliations:** † Soil Chemistry Group, 4508 Wageningen University & Research, P.O. BOX 47, Wageningen 6700 AA, The Netherlands; ‡ INVITE GmbH, Otto-Bayer-Straße 32, D-51061 Cologne, Germany; § Formulation Technology, 2022 ES Deutschland GmbH (ENVU), Alfred Nobel Str. 50, 40789 Monheim am Rhein, Germany

**Keywords:** pesticide, humic acid, goethite, competition, conformational change, surface complexation modeling

## Abstract

The environmental fate of the herbicide glyphosate (PMG) is determined by its favorable binding to metal (hydr)­oxides, which is affected by environmental factors and the presence of competitors. A major competitor binding to metal (hydr)­oxides is natural organic matter (NOM). This study investigated the competitive binding between humic acids (HA) and PMG on goethite with batch adsorption experiments, varying pH, ionic strength, and HA surface loading. HA strongly decreases PMG adsorption, increasing its solution concentration by multiple orders of magnitude. Interpretation of the competitive adsorption data with the NOM-CD model revealed that site and electrostatic competition insufficiently explain the competition. The model can be greatly improved by introducing steric hindrance as an additional mechanism, requiring only a single adjustable parameter. At low pH, HA maximizes its interaction with the surface while at high pH, the ligands tend to move outward. Our model reveals that steric hindrance is most significant in acidic conditions, while in alkaline conditions, competition is primarily controlled by electrostatics. The steric NOM-CD model provides excellent predictions of the behavior of PMG competing with HA and provides a tool to describe the key role of NOM in assessing the availability, mobility, and risk of PMG in the environment.

## Graphical abstract

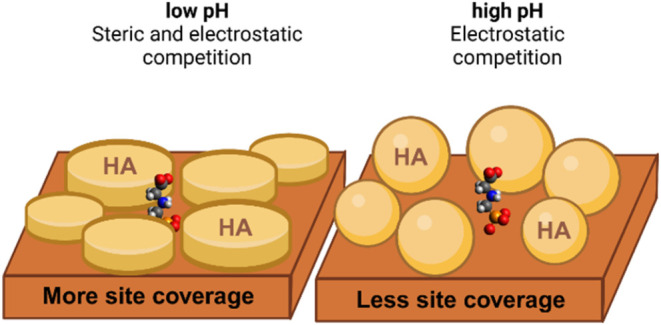

## Introduction

Glyphosate (N-phosphonomethyl glycine, or PMG) is a nonselective organophosphorus herbicide and is currently the most frequently used pesticide for weed control worldwide.^1,2^ Its widespread use is due to many factors,^3,4^ one of them is the strong sorption and supposed low mobility and risk of leaching to groundwater. Despite the assumed strong binding affinity, PMG residues are often found in groundwater^5^ and surface water.^2^ The adsorption behavior of PMG in soils, particularly the influence of soil composition and environmental conditions are to date poorly understood.

In soils and other natural systems, metal (hydr)­oxides are the primary adsorbents for PMG.^4,6–9^ Adsorption has been widely investigated, predominantly for goethite.^10–25^ For this crystalline Fe-oxide, a consistent surface-complexation model for PMG binding was recently developed in which PMG binds to goethite with its phosphonate group forming strong inner-sphere complexes in both bidentate and monodentate modes with both the distribution constant and surface speciation being highly dependent on pH.^26^

PMG is hydrophilic and anionic and has a low octanol–water partition coefficient (log *K*_ow_ = −6.28 ^27^), making hydrophobic interactions with NOM unlikely. In addition, since both NOM and PMG are primarily (net-) negatively charged, electrostatic binding can be excluded.

Despite the absence of strong interactions between PMG and NOM, NOM is a key factor for PMG partitioning in soils. NOM binds strongly to metal (hydr)­oxides^28–32^ thereby acting as a competitor to PMG binding on metal (hydr)­oxide surfaces^33,34^ and increasing its availability. Regression studies show a highly inconsistent relation between PMG sorption parameters in soils and the organic matter content, reporting positive,^35,36^ no,^35,37–39^ and negative^38,40^ correlations. Reported organic carbon normalized partition coefficients (*K*_oc_) for PMG vary over multiple orders of magnitude,^27^ indicating that the correlation between soil partition coefficients of PMG and organic carbon content is weak. While such empirical approaches can describe the sorption of PMG reasonably well for a single data set, the obtained regression parameters are not transferable to other soils and provide little mechanistic insight into the underlying processes.^41^ One complication of regression studies is the mutual correlation of soil parameters. Many studies find a positive correlation between the adsorption of PMG and the Fe- and/or Al-(hydr)­oxide content or the clay fraction, which generally contains most of the reactive Fe and Al minerals.^35–39^ Since the metal (hydr)­oxide content is in turn strongly correlated to the organic matter content,^42,43^ the importance of each soil constituent may be masked by the mutual interaction.

Various studies have been performed on simplified, synthetic systems to clarify the role of NOM in the sorption of PMG. Day et al.^34^ used freshwater NOM to coat synthetic goethite and observed a lower PMG adsorption than found for uncoated goethite. Arroyave et al.^33,44^ investigated the competitive adsorption of PMG and HA to goethite using Fourier-transformed infrared (FTIR) spectroscopy, confirming that HA has a strong inhibition effect on PMG adsorption, while PMG had no observable effect on adsorbed HA.

NOM interacts with metal (hydr)­oxides through multiple ligand exchange and outer-sphere complex formations.^31,45,46^ This complexation is central to the ligand and charge distribution (LCD) model that has been developed to predict the binding of NOM on goethite.^31^ This approach was recently extended to account for the heterogeneous nature of NOM and condition-dependent changes in the molecular conformation of interfacial NOM.^47^ For competitive systems and whole-soil applications, the natural organic matter-charge distribution (NOM-CD) model has been proposed.^29,48^ In this model, rather than describing the partitioning between dissolved and adsorbed NOM, NOM is only defined as a surface component with a given interfacial charge distribution, simulating the electrostatic interaction and site competition of adsorbed NOM. This approach has successfully been applied to HA or fulvic acid (FA) systems with oxyanions (PO_4_^3–^, AsO_4_^3–^, and As­(OH)_3_)^48,49^ and metal ions (Cu^2+^ and Cd^2+^).^50,51^ The NOM-CD concept is furthermore used in multisurface model applications.^52,53^

In the present study, the mechanistic principles of the competitive adsorption of PMG and NOM will be investigated. Batch adsorption experiments will be performed using HA as a proxy for NOM, varying pH, ionic strength, and HA loadings. The collected data will be interpreted using the NOM-CD model, which will subsequently be extended. This approach increases our insights into the underlying mechanisms that control the competition while simultaneously providing a mechanistic and practical surface complexation model, thereby contributing to a better understanding and prediction of the adsorption behavior and fate of PMG in natural systems.

## Material and Methods

### Primary Materials

Well-crystallized goethite (A_BET_ = 94 m^2^ g^–1^) was prepared and characterized in an earlier study.^54^ A goethite stock suspension of 29.9 g/L in 0.01 M NaNO_3_ was prepared and stored under N_2_ to avoid carbonation.

Humic acid was extracted from a forest soil in The Netherlands (Tongbersven) following an adapted protocol of the International Humic Substances Society (IHSS),^55^ adopted from Swift et al.,^56^ as detailed in the Supporting Information (SI) Section S1.

The final HA product had a carbon content of 0.422 g C g^–1^ HA, and an ash content of 6.3% w/w, containing 0.20 mol Al, 0.18 mol Fe and 0.34 mol Si per kg of HA, as determined by ICP-OES. Assuming all Al and Fe originate from Al- and Fe (hydr)­oxides (while the Si/Al ratio suggests that clay minerals may also contribute), these impurities would contribute less than 0.5% to the total (hydr)­oxide mass used in our experiments at the highest concentration of added HA. Since these minerals resisted dissolution in 0.1 M acid and base, the reactive surface area is supposedly small, and the contribution to the reactive oxide fraction will be negligible.

The charging behavior of our HA was characterized and interpreted using the potentiometric acid–base titration and model fitting procedure proposed by Tesfa et al.,^57^ adapted for the NICA-Donnan model^58^ (for details, see SI Section S2).

A 5.7 g HA L^–1^ stock solution was prepared by dissolving the freeze-dried HA in NaOH solution under N_2_ overnight, after neutralizing the solution with HNO_3_ and adjusting the ionic strength with NaNO_3_ to 0.1 M.

Glyphosate-isopropylamine stock solution (46% PMG), provided by Bayer, was used for preparing 1.45 and 1.64 mM PMG stock solutions in 0.1 M NaNO_3_ and 0.01 M NaNO_3_, respectively. The purity of the PMG stock was assessed using High-Performance Liquid Chromatography Mass Spectroscopy (HPLC-MS), which confirmedthe absence of any other compounds in observable amounts. Furthermore, total PMG concentration corresponded to the total P concentration measured using High-Resolution Inductively Coupled Plasma Mass Spectroscopy (HR-ICPMS, Thermo Scientific Element 2, limit of detection (LOD) = 0.03 μM P).

### Pre-Equilibration of the Goethite-HA Composites

For the PMG adsorption experiments, we first prepared goethite-HA composites by pre-equilibrating them at different HA and target pH levels, where we aimed for a high fraction of HA sorption to the goethite to minimize HA in solution. Composites were prepared at a high HA level (1.6 mg HA m^–2^ at pH 4, 5, and 6; 1.3 mg HA m^–2^ at pH 7 and 8), an intermediate HA level (1.1 mg HA m^–2^ at pH 4, 5, 6, 7 and 8), and a low HA level (0.5 mg HA m^–2^, at pH 4, 6, and 8). All composites were prepared in 0.1 M NaNO_3_. Additionally, the composites at the lowest HA level (0.5 mg HA m^–2^ at pH 4, 6, and 8) were repeated in a 0.01 M NaNO_3_ background, bringing the total number of systems to 16.

The composites were prepared by combining 20 mL of goethite stock suspension with a corresponding volume of HA stock solution, adding 1 M NaNO_3_, 0.1 M NaNO_3_, and water to reach the required ionic strength. The composites were placed in a horizontal shaker for 1 week to equilibrate and pH was adjusted daily using 0.1 or 0.01 M HNO_3_ and NaOH.

To reduce the excess volume due to pH and ionic strength adjustments, a day before the end of the equilibration time, the batches were centrifuged at high speed (18 000*g*) for 1 h and the excess volume of supernatant was removed to achieve a final volume of 30 mL, corresponding to a goethite concentration of 20.0 g L^–1^. The removed supernatant was analyzed for organic carbon with a Shimadzu C-analyzer (LOD = 0.3 mg L^–1^ C) to calculate the total HA concentration of the final composite suspension, corrected for the removed C. The precipitated goethite-HA composite was resuspended and shaken for the final day of equilibration before use in the PMG adsorption experiments.

### PMG Adsorption Experiments

For each pre-equilibrated HA-goethite system, a PMG isotherm was determined. In 1.5 mL centrifuge vials, 0.5 mL of the composite suspension was added. Subsequently, the PMG stock solution of the corresponding ionic strength was added to the suspension (0.50, 0.40, 0.35, 0.30, 0.25, 0.20, 0.15, 0.10, and 0.05 mL). The volume was adjusted to 1 mL with 0.1 or 0.01 M NaNO_3_, yielding a final goethite concentration of 10.0 g L^–1^. In addition, a blank HA-goethite system without PMG addition was prepared for each composite by adding 0.5 mL of the composite to 0.5 mL of background solution. The pH was not further adjusted in our systems, causing some deviation from the target pH (Table S4). The pH variation of the systems within each isotherm was limited to ≤ 0.3. In our modeling calculations, this variation was accounted for by using the individual experimental pH values.

The HA-goethite systems with the added PMG were equilibrated in a horizontal shaker for a reaction time of 24 h, reaching near-equilibrium as no further decrease in the measured PMG solution concentrations occurred. Subsequently, the samples were centrifuged at high speed (18 000*g*) for 45 min. An aliquot of 0.5 mL supernatant was diluted 1:20 with 0.14 M HNO_3_ in which PMG was determined by measuring phosphorus (P) with HR-ICP-MS. The pH was measured in the remaining sample suspension, using a micro pH electrode (Orion ROSS Sure-Flow Thin Stem pH electrode, ThermoFisher).

The blanks were analyzed for DOC by taking 0.5 mL from the supernatant and diluting 1:20 with water. An additional aliquot of 0.1 mL from the blanks was diluted 1:100 for P measurement with HR-ICP-MS, which were consistently below the LOD, indicating no measurable contribution of P from HA in solution. Because PMG contributes to the DOC concentration, we did not measure DOC in the systems with PMG. Instead, we used the experimental NOM loading of the blank for all systems of the same composite, since PMG has little effect on adsorbed HA.^33^

### PMG Surface Complexation Model

For modeling the PMG interaction with goethite, we used the CD model.^59^ The charge of adsorbed PMG was distributed in the compact layer of the electrical double layer (EDL) defined by the extended Stern layer approach. Singly (≡FeOH^–0.5^) and triply (≡Fe_3_O^–0.5^) coordinated surface groups with corresponding site densities were distinguished for goethite.^60^ The specific surface area, point of zero charge (PZC), and capacitance values of our goethite were determined previously.^54^ Affinity constants and CD coefficients of H^+^, Na^+^, and NO_3_^–^ were taken from Hiemstra et al.^61^ and those for the various PMG surface complexes, derived for the same goethite, from Geysels et al.^26^ The PMG solution speciation is given in Table S2, and all adsorption parameters are given in Table S3.

### NOM-CD Model

The principles of the NOM-CD model are described elsewhere in detail.^29,48^ In short, the NOM-CD model introduces a NOM surface component ≡HNOM^–1^, representing two carboxylic groups of which one is protonated, to mimic both the electrostatic and site competition effects of interfacial NOM. Since the model does not consider the partitioning of NOM between the solution and the goethite surface, the ≡HNOM^–1^ surface density is an input parameter for the model.

In the NOM-CD model, the ≡HNOM^–1^ surface component reacts with a singly coordinated goethite surface site (≡FeOH^–0.5^), forming three possible surface species: a nonprotonated (≡FeNOM^–1.5^) and a protonated (≡FeNOMH^–0.5^) inner-sphere complex, and an outer-sphere complex (≡FeOH_2_^+0.5^-NOM^–2^). At the formation of these species, the sum of the charge of the surface components ≡HNOM^–1^ and ≡FeOH^–0.5^ (−1.5 v.u.) is redistributed over the electrostatic planes by defining charge distribution (CD) coefficients. The relative contribution of the three NOM surface species is determined by their intrinsic affinity (log *K*) and electrostatic energy acting on the distributed charges in the Stern layer (see Table 1).

**Table 1:** Illustrative surface speciation table for the S-NOM-CD model concept, defining a theoretical system with solely protons as aqueous species, including the surface reactions of H^+^, with ≡FeOH, ≡HNOM, and ≡S^0^ surface components.^a^

complex	≡FeOH^–0.5^	≡HNOM^–1^	≡S^0^	Δ*z*_0_	Δ*z*_1_	Δ*z*_2_	H^+^	log *K*
≡FeOH^–0.5^	1	0	0	0	0	0	0	0
≡FeOH_2_^+0.5^	1	0	0	1	0	0	1	9.3^d^
≡FeNOM^0,‑1,‑0.5^	1	1	0	1.5	–1.0	–0.5	0	0
≡FeNOMH^0,‑0.5,0^	1	1	0	1.5	–0.5	0	1	log *K*_H_ ^b^
≡FeOH_2_–NOM^+0.5,‑1.5,‑0.5^	1	1	0	2	–1.5	–0.5	0	0.6^c^
≡S^0^-FeOH^–0.5^	1	0	1	0	0	0	0	0
≡S^0^-FeOH_2_^+0.5^	1	0	1	1	0	0	1	9.3^d^

a The full surface speciation database, used for the model calculations of our PMG and HA systems, is given in Table S3.

b Value depends on the type of NOM material used. For our HA, the value is from Deng et al.^49^

c Value taken from Hiemstra et al.^48^

d PZC of our goethite, as determined by Weng et al.^54^

The overall surface site density of ≡HNOM, defined by the sum [≡FeNOM] + [≡FeNOMH] + [≡FeOH_2_–NOM], can be found by fitting the calculated adsorption of a competitor (e.g., PO_4_^3–^, AsO_4_^3–^) to the measured value.^48,49^ In earlier applications of the NOM-CD model to HA-oxyanion systems, it was shown that the fitted ≡HNOM surface density is proportional to the amount of HA adsorbed.^48,49^ We will use this observation to constrain our model when describing the PMG adsorption isotherms at different pH and HA surface loadings, by relating the ≡HNOM surface density linearly to a relative amount of bound HA (Γ_NOM_/Γ_ref_), according to

1 $$\left[\equiv \mathrm{HNOM}\right]=\left(\frac{\Gamma_{\mathrm{NOM}}}{\Gamma_{\mathrm{ref}}}\right)\times \left[\equiv \mathrm{FeNO}M_{T}\right]$$

where Γ_NOM_ is the experimental NOM loading (Table S4), and [≡FeNOM_T_ ] is the total ≡HNOM surface density at a chosen reference NOM loading Γ_ref_, which, conform with earlier application of the NOM-CD model, is chosen to be the maximum HA loading in our experiments (Γ_ref_ = 1.6 mg HA m^–2^) so­[≡HNOM] will reach the value of [≡FeNOM_T_ ] at Γ_NOM_/Γ_ref_ = 1. The value ≡FeNOM_T_ expresses the competitive strength of a given HA and, at equal NOM loadings, is linearly related to the carboxylic density (*Q*_RCOO_) of the NOM,^48,49^ which will be used in our interpretation of the PMG competition with HA, as detailed in the Results and Discussion section.

### Software and Parameter Optimization

The surface complexation model was implemented in ORCHESTRA^62^ coupled with PEST^63,64^ for parameter estimation. Parameters (≡FeNOM_T_ and/or ≡S^0^, see Results and Discussion) were optimized by minimizing the root-mean-square error (RMSE) between the modeled and observed logarithm of the PMG solution concentration (M) of our data set (*n* = 104).

## Results and Discussion

### Pre-Equilibrated HA-Goethite Composites

The final HA loading in our experiments varied from Γ_NOM_ = 0.5–1.6 mg HA m^–2^ goethite (Table S4), well below the maximum adsorption of HA which is typically ∼3 mg HA m^–2^ for goethite at low pH.^32^ Our loadings are representative for the competitive loading of mineral-associated NOM in temperate and tropical agricultural top soils, which range around ∼0.5–2 mg NOM m^–2^.^48,52^

While NOM prefers binding within the compact part of the electric double layer (EDL),^47,48^ part of the HA may enter the diffuse double layer region.^29,30^ A Stern layer of ∼0.8 nm thickness can accommodate ∼1 mg NOM m^–2^, at an assumed mass density of 1250 kg m^–3^.^32,65^ In our experiments, HA at low loading can be fully accommodated in the Stern layer, only occupying about half of the Stern layer volume. At our highest loadings, part of the HA cannot be accommodated in the Stern layer volume and must be located in the diffuse double layer.

In agricultural topsoils, the NOM layer thickness on metal (hydr)­oxides varies between ∼1–3 nm,^42^ indicating that in natural systems, a considerable part of the mineral-associated organic carbon is bound outside the compact part of the EDL, with only a fraction being directly involved in the competition with other adsorbates that bind more closely to the surface.^66^

### Competitive Adsorption of HA and PMG

The PMG adsorption is high (>70%) in all experiments due to the relatively high goethite to PMG ratio used. At low pH, dissolved PMG concentrations were often below the LOD of the HR-ICP-MS. For the lowest total HA concentration, PMG solution concentrations for the isotherms at pH 4 were all below LOD and are therefore not reported. The measurable solution concentrations ranged from 6·10^–7^–2·10^–4^ M, the lower end (10^–6^–10^–7^ M) is typically found in surface and pore waters of agricultural areas where PMG is applied.^67,68^

Figure 1 shows the isotherms at the different HA levels and varying pH and ionic strength on a log–log scale. Here, the slope reflects the degree of nonlinearity. All PMG adsorption isotherms show a clear nonlinearity, only approaching a slope of ∼1 at low PMG loading. The PMG adsorption furthermore shows a strong dependence on both pH and HA loading.

**Figure 1:**
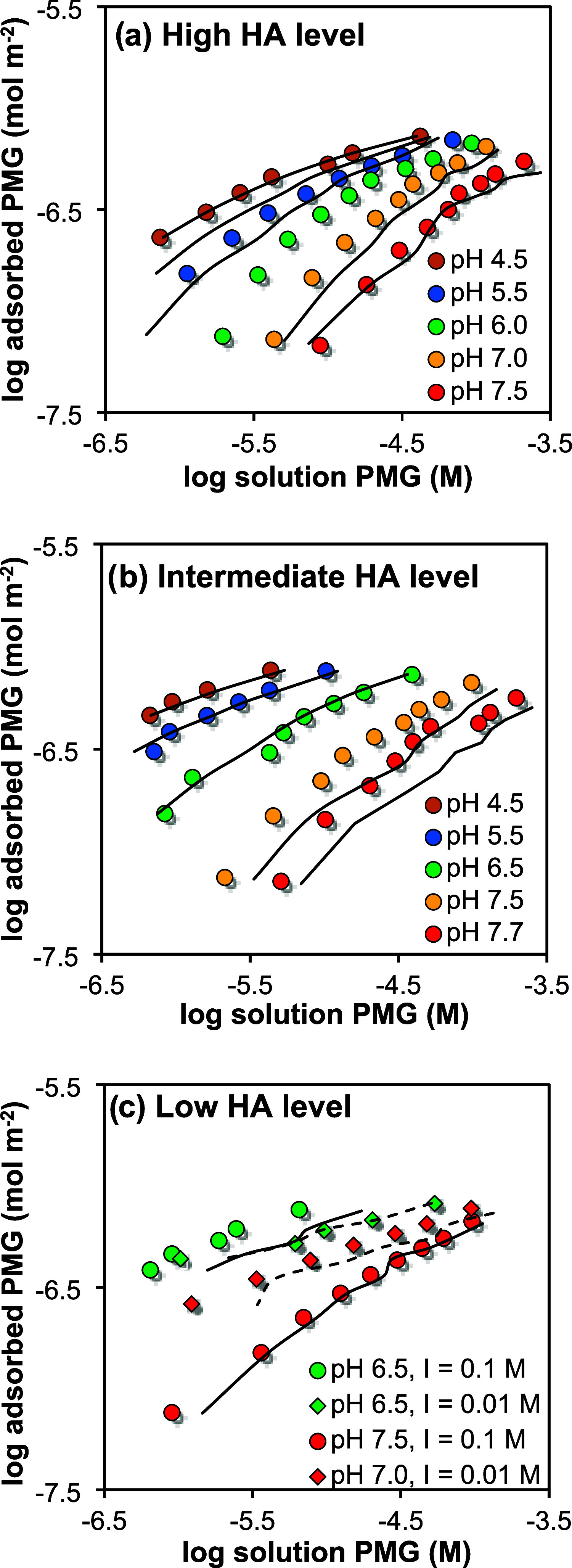
PMG adsorption isotherms of competitive HA-PMG systems in 10.0 g goethite L^–1^ (94 m^2^ g^–1^) in 0.1 M NaNO_3_ (circles and solid lines) and 0.01 M NaNO_3_ (diamonds and dashed lines). Lines represent Steric-NOM-CD model calculations (see text) using the parameters listed in Table S3. A fixed value of ≡FeNOM_T_ (1.77 μmol m^–2^) was used with ≡S_max_^0^ = 2.56 ± 0.14 (0.95 CI) μmol m^–2^ being fitted as the only optimized parameter (r^2^ = 0.964 and RMSE = 0.197). Total HA levels are (a) 1.6 mg HA m^–2^ for pH 4.5, 5.5, and 6.0, and 1.3 mg HA m^–2^ goethite for pH 7.0 and 7.5, (b) 1.1 mg HA m^–2^, and (c) 0.5 mg HA m^–2^. The corresponding HA surface loadings can be found in Table S4. The pH for each isotherm provided in the legend represents the average experimental pH value, with a variation of ≤ 0.3 pH units. The model lines have been calculated using the individual pH values for each data point, resulting in a nonsmooth result. A point-by-point comparison of the experimental and modeled results is provided in Figure S4.

HA has a strong impact on the binding of PMG. Figure 2 shows the solution concentration of PMG as affected by different levels of HA for the highest PMG level used in our experiments, compared to the modeled solution concentration in the absence of HA for reference. The modeled isotherms in absence of HA as compared to the experimental data are provided in Figure S2. Both figures show that the effect of HA is largest at low pH, increasing PMG concentrations in solution by up to 3 orders of magnitude for the high HA loading, while at neutral pH, the effect is limited. A similar behavior is found for phosphate, attributed to a change in the location of the reactive NOM ligands in the interface.^66^ However, this is also a result of scaling. At an equal desorption of PMG, the logarithm of the solution concentration changes less when the solution concentration is already high.

**Figure 2:**
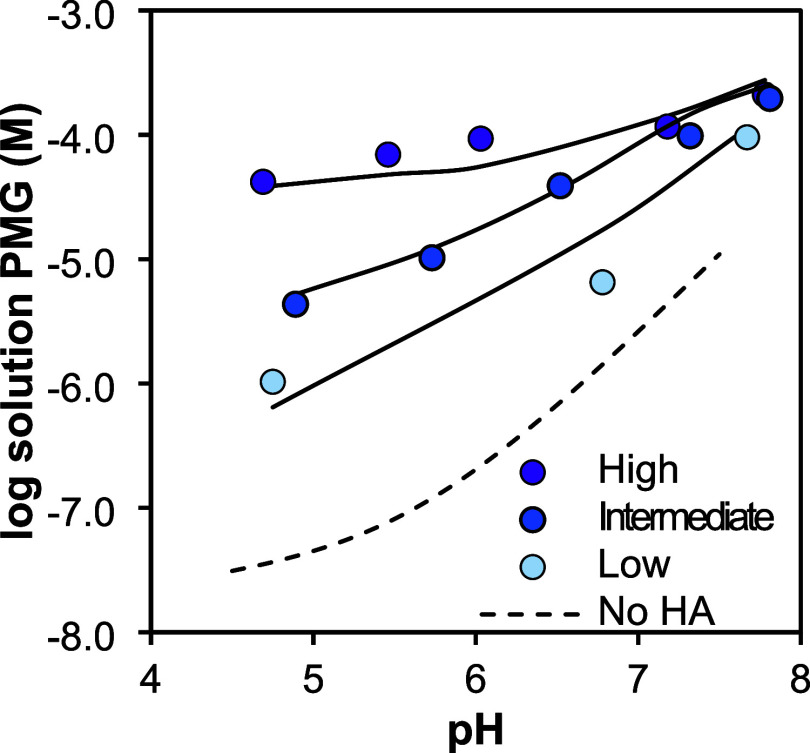
Logarithm of the solution concentration of PMG as a function of pH for different HA levels at a total PMG concentration of 0.72 mM for 10.0 g L^–1^ (94 m^2^ g^–1^) goethite systems in 0.1 M NaNO_3_. The high HA systems contain 1.6 mg HA m^–2^ for pH 4.7, 5.7, and 6.0, and 1.3 mg HA m^–2^ goethite for pH 7.2 and 7.8. The intermediate HA level is 1.1 mg HA m^–2^, and the low HA level is 0.5 mg HA m^–2^. Solid lines represent the Steric-NOM-CD model predictions using the parameters listed in Table S3, and the dashed line is the modeled PMG solution concentrations in the absence of HA (see text).

Ionic strength had no strong impact on the HA loading in our systems (Table S4). The PMG sorption is reduced at a lower ionic strength for pH ∼6.5, indicating stronger competition by HA, likely through stronger electrostatic repulsion (Figure 1). For the highest pH (∼7.5), PMG sorption is, in contrast, slightly higher at low ionic strength. However, this is more likely related to the 0.5 unit lower final pH (∼7.0) in the treatment with a low ionic strength. This will be later confirmed by model calculations (see Section Application of the S-NOM-CD model).

### Surface Density of ≡FeNOM_T_

The surface density ≡HNOM is a crucial parameter in modeling the competition of PMG and adsorbed HA. For small oxyanions, the total density of the ≡HNOM surface species has been derived by fitting the calculated oxyanion adsorptions to the experimental values. We will calculate this value *a priori*, as it reduces the number of free parameters and may lead to insights into the competition of NOM. As mentioned in the section Material and Methods, the competitive strength of HA and FA, represented by ≡FeNOM_T_, is linearly related to the carboxylic group density (*Q*_RCOO_) of NOM materials at equal NOM loadings.^48,49^ By normalizing the reported ≡FeNOM_T_ to the corresponding reference NOM loading (Γ_ref_), we quantified and generalized this relationship. For a direct comparison to the carboxyl density of the various HAs and FAs, ≡FeNOM_T_ can additionally be recalculated to the number of carboxyl groups involved by multiplication with a factor of 2, as each ≡FeNOM_T_ species represents two RCOO^–^ groups, yielding

2 $$\left[\equiv {\mathrm{FeNOM}}_{T}^{*}\right]=2\frac{\left[{\equiv \mathrm{FeNOM}}_{T}\right]}{\Gamma_{\mathrm{ref}}}$$

The normalized ≡FeNOM_T_* has the unit mol RCOO^–^ kg^–1^ NOM and represents the amount of surface associated carboxylates at the used reference NOM loading. It can be directly compared to the carboxylic site density (*Q*_RCOO_) of the NOM materials applied in the various competition experiments (Figure 3). A linear relationship is found with an almost 1:1 ratio between both quantities, indicating that in the underlying experiments, nearly all carboxylate groups of the adsorbed NOM are directly associated with the goethite surface (Figure 3).

**Figure 3:**
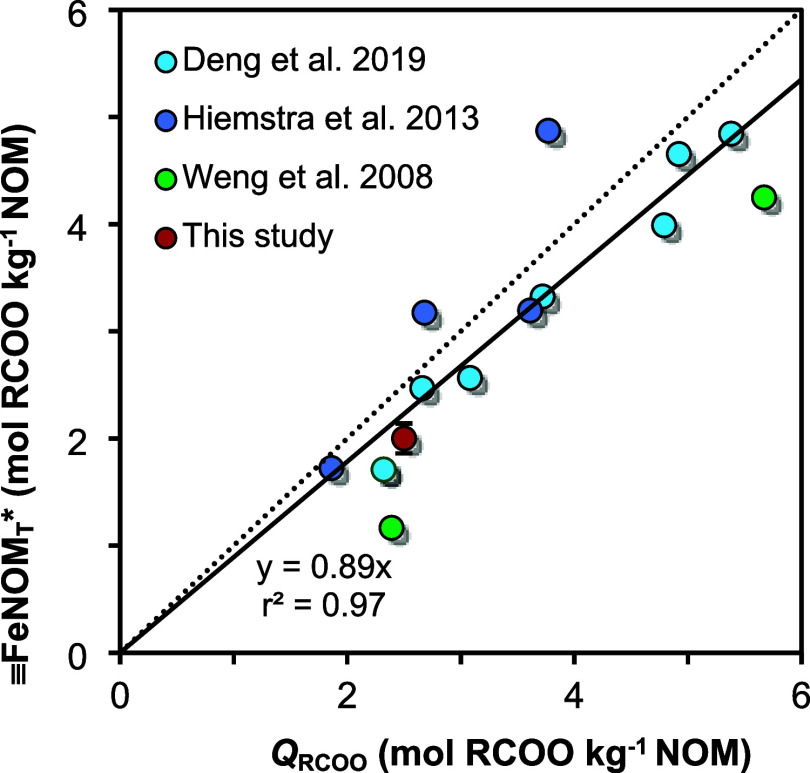
Relationship between the surface-associated carboxylates (≡FeNOM_T_*), calculated according to eq 2, and total carboxylate density (*Q*_RCOO_) of different NOM materials, showing a nearly 1:1 relationship (dotted line). The data were retrieved from Hiemstra et al.,^48^ Deng et al.,^49^ and Weng et al.^69^ The relationship ≡FeNOM_T_* = 0.89 ± 0.10 × *Q*_RCOO_ (0.95 CI; solid line) was used to calculate ≡FeNOM_T_ for our system. The red circle represents the result obtained in this study by freely fitting ≡FeNOM_T_ in our S-NOM-CD model (see section Application of the S-NOM-CD model), but was not included for the linear regression.

Using the linear relationship in Figure 3 and applying eq 2, one can calculate the expected value for ≡FeNOM_T_ being 1.77 ± 0.20 (0.95 CI) μmol m^–2^ for our HA with *Q*_RCOO_ = 2.5 mol RCOO^–^ kg^–1^ (Table S1). This value can be used to calculate the surface density [≡HNOM] for each data point using eq 1, to be applied in the NOM-CD modeling.

### Basic NOM-CD Modeling

The above value for ≡FeNOM_T_ was used in the NOM-CD model to predict PMG adsorption in the presence of HA. At high pH, the competitive PMG adsorption is generally well predicted (Figure S3). In addition, the PMG predictions are reasonable at low NOM loading. This suggests that the competition at these conditions is similar to that found for oxyanions. The latter behavior is primarily driven by site and electrostatic competition. On the contrary, the basic NOM-CD model drastically overpredicts the PMG adsorption at low pH (Figure S3). Free fitting of the parameter ≡FeNOM_T_, rather than using the linear relationship to *Q*_*RCOO*_ (Figure 3), could not provide a satisfactory description of the competitive adsorption data on both low and high pH.

It suggests that the competition under these conditions is controlled by an additional factor that can strongly suppress the PMG binding to goethite in the presence of HA. An important difference between inorganic anions and PMG is the adsorption volume. For PMG, this may lead to physical constraints when voluminous HA (supra) molecules^70^ occupy the compact part of the EDL, covering reactive sites for PMG. When adsorbed, PMG molecules tend to stretch out, occupying the full Stern layer domain.^26^ In contrast, the much smaller oxyanions are partly incorporated into the surface, enabling binding while covered by adsorbed HA. For the adsorption of FA, space limitation has also been suggested as an important factor in the competitive interaction with adsorbed HA.^71^

Current views consider HA to be a supramolecular association of individual molecules with an overall soft and penetrable rather than a rigid structure.^70,72–74^ When bound to an oxide surface, HA alters its conformation.^32,48,65^ Recent insights using an advanced version of the ligand and charge distribution (LCDcc) model^47^ suggest that HA preferentially occupies the Stern layer at conditions that are favorable for inner-sphere complexation of the carboxylic groups. At these conditions, the pH and HA loadings are low, and the electrostatic field is strongly attractive. The strong interaction will lead to a change in the molecular conformation of the supramolecular HA particles. At an increased pH, the molecular structure can relax and the reactive ligands of adsorbed HA tend to move outward and, at high loading, into the diffuse double layer, uncovering surface sites.^47^ This may allow PMG to adsorb with fewer physical restrictions. To account for the process of physical space limitation, we have developed a steric NOM-CD model, as presented next.

### Steric NOM-CD Model

The concept of space limitation can be implemented in the NOM-CD model, yielding the steric NOM-CD (S-NOM-CD) model. By introducing a zero-charged surface component, ≡S^0^, that can react with singly coordinated surface sites, the site is made unavailable for binding with PMG. The corresponding ≡S^0^-FeOH sites remain available for all other ions that are not sterically hindered. For our system, containing H^+^, Na^+^, NO_3_^–^, the formation of ≡S-FeOH^–0.5^, ≡S-FeOH_2_^+0.5^, ≡S-FeOH^–0.5^-Na^+^, and ≡S-FeOH_2_^+0.5^-NO_3_ are defined with equal affinity constants and CD coefficients as the corresponding species formed from regular ≡FeOH^–0.5^ sites. Similarly, one can define, if applicable, other ≡S-species for nonsterically hindered ions, for instance, a set of ≡S-PO_4_ surface species. The introduction of the subset of singly coordinated sites will not influence the primary charging of the surface and adsorption characteristics in the absence of steric hindrance. The ≡S^0^ surface density, similarly to ≡HNOM, is an input parameter for the model, where [≡S^0^] equals the sum of all ≡S-species.

The defined concept is illustrated in a surface speciation table (Table 1) for a theoretical S-NOM-CD system with solely aqueous protons reacting with the surface. The complete speciation table for the PMG-HA system as used in our model calculations is provided in Table S3.

A central question in the modeling of the PMG adsorption is the number of sterically hindered sites, i.e., [≡S^0^]. The value will depend on the molecular conformation of adsorbed HA. A first attempt to describe conformational changes of adsorbed HA was made by Xu et al.^32^ The authors limited the maximum occupation of the Stern layer space by adsorbed HA and defined the condition-dependent maximum allowed HA adsorption in the Stern layer space (Γ_MST_) relative to the physical maximum (Γ_MST_^0^), according to

3 $$\theta_{S}=\frac{\Gamma_{\mathrm{MST}}}{\Gamma_{\mathrm{MST}}^{0}}$$

in which θ_S_ represents the maximum allowed degree of filling of the physical Stern layer space, and the physical maximum adsorbed Γ_MST_^0^ = 1 mg NOM m^–2^.^32^ At favorable binding conditions i.e., low pH, near-saturation (θ_*S*_ ∼ 1) of the Stern layer space with HA is possible, whereas at neutral conditions, only about half of the Stern layer can be occupied by HA (θ_S_ ∼ 0.5) due to changes in the molecular conformation.^32^ To describe the variation in θ_S_ as a function of solution conditions, Xu et al.^32^ have introduced an empirical relation with the proton activity (H) and ionic strength (*I*) as variables, according to

4 $$\theta_{S}=\frac{K{\left(H\right)}^{a}I^{b}}{1+K{\left(H\right)}^{a}I^{b}}$$

where *K*, *a*, and *b* are empirical coefficients equaling *K* = 80, *a* = 0.32 and *b* = −0.33.

The concept of a conditional maximum Stern layer occupation (θ_S_) can be used in the S-NOM-CD model by assuming proportionality of the steric surface component density (≡S^0^) with θ_S_, reaching a maximum value (≡S_max_^0^) at θ_S_ = 1, according to

5 $$\left[{\equiv S}^{0}\right]=\theta_{S}\left[\equiv S_{\max}^{0}\right]$$

In our modeling, we will implement the conditional value of θ_S_ (eq 4) into the relation of eq 5, making the value of ≡S_max_^0^ the only free adjustable parameter.

### Application of the S-NOM-CD Model

The above S-NOM-CD model has been parametrized, using ≡FeNOM_T_ with a value of 1.77 μmol m^–2^ as determined above (see Surface Density of ≡FeNOM_T_). This yields for ≡S_max_^0^ a value of 2.56 ± 0.14 (0.95 CI) μmol m^–2^. The PMG adsorption isotherms calculated with the parametrized model are shown in Figure 1. A good agreement is found between the modeled and experimental data (r^2^ = 0.964 and RMSE = 0.197), with major improvements of the model predictions toward low pH and increasing Stern layer occupation compared to the basic NOM-CD model. The developed S-NOM-CD model can predict the competitive effect on the binding of PMG using only a single adjustable parameter.

We have used the model approach to verify the applied value for ≡FeNOM_T_, by treating it as an additional adjustable parameter. Parameter optimization of this two-parameter model yields ≡S_max_^0^ = 3.04 ± 0.33 (0.95 CI) μmol m^–2^ and *≡*FeNOM_T_ = 1.60 ± 0.11 (0.95 CI) μmol m^–2^ (r^2^ = 0.963 and RMSE = 0.189). This value fitted for ≡FeNOM_T_ is, within the uncertainty range, equal to the value derived from the normalized proportionality relation of Figure 3.

As evident from comparing Figures 1 and S3, the difference between modeling with the basic and S-NOM-CD model is small at high pH. At these conditions, the impact of steric hindrance is limited, and the basic NOM-CD model already provides a good prediction, without fitting. This can be understood by considering a system at pH 7 with an ionic strength of *I* = 0.1 M. At these conditions, the maximum degree of Stern layer saturation will be θ_S_ ∼ 50% (eq 4), which translates for PMG to a Stern layer space occupation by HA of ≡S^0^ = ∼1.3 μmol m^–2^ or ∼0.8 sites nm^–2^ (eq 5). This implies that only ∼25% of the total site density of singly coordinated groups (3.45 sites nm^–2^) is blocked by steric hindrance, which has a limited impact on the PMG adsorption. At these conditions, the PMG adsorption is primarily electrostatically controlled. Here, the S-NOM-CD model underpredicts the PMG adsorption slightly at high pH for most treatments, which we discuss in the section Physicochemical Interpretation.

At low pH, the S-NOM-CD and basic NOM-CD model predictions deviate strongly (Figures 1 and S3, respectively). Using similar calculations for pH 4.5, this can be understood from the high θ_S_ value of ∼85% leading to ≡S^0^ ∼2.2 μmol m^–2^ or ∼1.4 sites nm^–2^, which is equivalent to a reduction of ∼40% of the available singly coordinated sites due to steric hindrance. In addition, sites are consumed by direct surface interactions of NOM. At high NOM loading, ≡HNOM = ∼ ≡FeNOM_T_ = 1.1 sites nm^–2^, i.e., an additional ∼30% of the ≡FeOH sites is involved in a direct chemical binding, making the total number of nonavailable sites (∼70%) significant and crucial for regulating the PMG sorption at these conditions.

The model deviates from the experimental values at some instances at low PMG solution concentrations. This deviation is not consistent, but the magnitude of the deviations is likely due to analytical variation, as these PMG solution concentrations are close to the LOD, which is further magnified due to error propagation in the correction for dilution rates.

To confirm that the apparent contrasting ionic strength effect in our observations (Figure 1c) is in fact due to variation in pH between the isotherms, we used our model to calculate the isotherms at fixed pH (Figure S5). The calculations show that, at the same pH, PMG sorption is consistently lower at decreased ionic strength. This can be attributed to a higher electrostatic repulsion at low ionic strength, and potentially to the higher Stern layer occupation of HA at low ionic strength (see eq 4).

### Physicochemical Interpretation

The ligand and charge distribution in the Stern layer domain can vary significantly. According to a recent LCDcc approach,^47^ the fraction of charged carboxyl groups in the first Stern layer is *R* = 0.5 at a large potential gradient (Δψ_0–1_) typical for a low pH. This value of *R* is in line with the structure of the ≡FeNOM^0,‑1,‑0.5^ species, composed of two RCOO^–^ groups of which half is located in the first Stern layer forming an inner-sphere complex and the other half is in the second Stern layer. At high pH, outer-sphere complexation is relatively important. The outer-sphere species in the NOM-CD approach (≡FeOH_2_–NOM) have a structure with no carboxylic groups occupying the first Stern layer. This structure is equivalent to the ratio *R* = 0. According to the LCDcc modeling of Xu et al.,^47^ the distribution ratio of charged groups (*R*) decreases to nearly zero at very low values of the potential gradient (Δψ_0–1_), typically for high pH values near the PZC.

With the S-NOM-CD model, the ratio of inner- and outer-sphere complexation can be derived and translated into a carboxyl group distribution over the first and second Stern layer (*R*). Speciation calculations show a contribution of ∼2.5% outer-sphere NOM complexes at low pH and high HA loading, corresponding to *R* ∼0.49. At pH 7.5 and low HA loading, this contribution increases to ∼35% outer-sphere, or *R* ∼0.32. Theoretical calculations assuming a Γ_NOM_/Γ_ref_ = 0.1 at pH of 10 and pH 12 yield ∼75% and ∼90% outer sphere complexes, corresponding to *R* ∼0.10 and *R* ∼0.05, respectively. These calculations illustrate that the NOM-CD model has the ability to describe the switch of carboxylic groups in the compact part of the EDL revealed with recent advanced LCD modeling.

The empirical relation eq 4 describes the Stern layer occupation in function of pH and I, the latter two parameters essentially determine the electrostatic potential of the goethite surface.^47^ In our systems, the potential and consequent ligand distribution of NOM within the Stern layer is further affected by adsorbed PMG. At low pH and high HA loading, the contribution of outer-sphere NOM complexes is rather constant regardless of the PMG concentration (<0.1% variation). However, at high pH, increasing PMG concentrations significantly reduce the contribution of outer-sphere NOM complexes (up to 5% variation). This indicates that, while PMG does not affect the amount of NOM adsorbed,^33,44^ it could affect its conformation, in particular at high pH. This might explain the overprediction of the steric effect at high pH, as potentially, the negative electrostatic potential of adsorbed PMG causes the NOM to move away from the surface thereby reducing further space limitation, an effect that is not accounted for in our model.

An aspect that was not studied for competitive sorption with PMG is the variability of NOM of different types and compositions. Similarly to ≡FeNOM_T_ which varies with different NOMs in line with their *Q*_RCOO_,^48,49^ the relationship between the wider (supramolecular) properties of NOM and ≡S_max_^0^ can be part of future exploration with the S-NOM-CD model.

### Maximum Stern Layer Loading

Our data evaluation of the competitive adsorption of HA and FA with oxyanions (Figure 3) suggests that the vast majority (∼89%) of the carboxylic groups of adsorbed NOM materials are located close to the surface in the Stern layer domain. This relation is derived for systems with NOM loadings lower than the maximum Stern layer occupation of 1 mg NOM m^–2^.^48,49^ Our data included experiments with higher HA loadings up to 1.6 mg HA m^–2^ (Figure 1a). From a volume perspective, this high amount cannot be fully accommodated in the Stern layer considering the calculated physical maximum Stern layer occupation of Γ_MST_^0^ = 1 mg NOM m^–2^. It could suggest that a considerable part of the 1.6 mg HA m^–2^ is located outside of the Stern layer domain, while the value for ≡FeNOM_T_ suggest still ∼89% of carboxylates is located close to the surface. This would imply that the charged carboxyl groups may preferentially enter the Stern layer domain at favorable conditions. This observation is in line with the molecular modeling observations of Gerzabek et al.,^73^ who showed that the supramolecular nature allowed NOM to orientate its hydrophilic, oxygen-rich groups toward a positively charged metal (hydr)­oxide surface. Another factor contributing to this apparent disproportional presence of carboxyl groups near the surface could be the assumed mass density of the adsorbed HA (ρ = 1250 kg m^–3^) used in calculating Γ_MST_^0^.^32^ Potentially, the carboxylate groups are tightly bound to the surface, driven by inner-sphere complexation, and the local structure of adsorbed HA may be more condensed than suggested by the applied density.

### Environmental Significance

Glyphosate adsorption to metal (hydr)­oxides, the dominant process controlling the retention of the pesticide in soils and sediments, is strongly impacted by competition with NOM. The competition can increase the solution concentrations of glyphosate by multiple orders of magnitude. This competitive interaction is an important process that can contribute to enhanced mobility and transport of glyphosate, polluting surface- and groundwaters contrary to glyphosate’s supposed immobility.

For an accurate prediction of glyphosate behavior in natural systems, it is essential to account for the steric hindrance of NOM in the adsorption process. While the basic NOM-CD model can describe the influence of NOM on the competitive adsorption of oxyanions, it comes short for relatively large organic molecules such as glyphosate. By developing our S-NOM-CD model, for the first time, the behavior of glyphosate in a natural organic matter-metal (hydr)­oxide system can be quantified, enabling excellent predictions of the glyphosate concentration in solution. The S-NOM-CD model can be extended seamlessly by including the oxyanions and describing the competitive role of phosphate. Its applicability can be further expanded by introducing the cooperative interaction of divalent cations, thereby enabling whole-soil applications. Additionally, the model has the potential to be applied to other larger-sized organic chemicals that bind metal (hydr)­oxides such as pharmaceuticals and PFAS.

This study contributes to a more general understanding of NOM-mineral interactions. We reported evidence for a supramolecular conformational change to allow the preferential orientation of the carboxylate functional groups to the oxide surface and conformational-dependent space limitation within the Stern layer. Our framework provides insight into the processes of electrostatic, site, and steric competition, and can describe this complex process with a practical, yet mechanistically sound model with a minimum number of adjustable parameters.

## Supplementary Material



## Data Availability

The data and ORCHESTRA code will be made available at: 10.4121/5031018e-5d72-4757-90f0-45e0cd1fdc34.

## Notes

Isolation and proton titration of HA; thermodynamic database for S-NOM-CD modeling; pre-equilibrated HA-goethite composites details; effect of HA on PMG adsorption model calculations; model results using the basic NOM-CD model; point-by-point comparison of final model results; theoretical calculations confirming the ionic strength effect (PDF)

## ∥

Héctor S. Apreza Arrieta - Université Paris Cité, Institut de physique du globe de Paris, CNRS, Paris F-75005, France - apreza@ipgp.fr
