# Supplementary material for: Steric Hindrance of Glyphosate Adsorption to Metal (Hydr)oxides: A Novel Model Approach for Organic Matter-Mineral Interactions
Source: Environ Sci Technol. 2025 Jul 7;59(27):14020–9. doi: 10.1021/acs.est.5c04207 (PMC12269068; doi:10.1021/acs.est.5c04207)
Supplement: Supplementary file 1 [file es5c04207_si_001.pdf]

Supporting information

Steric hindrance of glyphosate adsorption to metal (hydr)oxides: A novel model approach for organic matter-mineral interactions

Bram Geysels, [a,b] Jan E. Groenenberg,[a] Tjisse Hiemstra,[a] Héctor S. Apreza Arrieta,[a†] Arnoldus W.P. Vermeer,[c] Rob N. J. Comans [a]*

[a]Soil Chemistry Group, Wageningen University & Research, P.O. BOX 47, Wageningen 6700 AA, The Netherlands

[b]INVITE GmbH, Otto-Bayer-Straße 32, D-51061 Cologne, Germany

[c] Formulation Technology, 2022 ES Deutschland GmbH (ENVU), Alfred Nobel Str. 50, 40789 Monheim am Rhein, Germany

*corresponding author: bram.geysels@wur.nl

Summary:

12 pages

5 figures

4 tables

Table of contents

Table of contents 3

S1 Humic acid isolation and proton titration 4

S2 Thermodynamic database 6

S3 HA-goethite composites 9

S4 Effect of HA on PMG adsorption 10

S5 Model results using basic NOM-CD 11

S6 Point-by-point comparison of model results...13

S6 Effect of ionic strength 14

References...15

S1 Humic acid isolation and proton titration

Humic acid isolation

Humic acid was extracted from a forest soil in the Netherlands (Tongbersven) following an adapted protocol of the International Humic Substances Society (IHSS),[1] adopted from Swift et al.[2] In short, 2 mm sieved soil was washed with 0.1 M HCl to remove metal ions and acid-soluble NOM, after which the HA fraction was extracted with 0.1 M NaOH under N_2 for 18h followed by centrifugation. The HA in the supernatant was precipitated by acidifying the extract to pH 1. The precipitate was redissolved in 0.1 M NaOH under N_2. Here, the protocol deviates from the standard IHSS protocol, as we have avoided HF treatment which can lead to chemical changes in the HA.[3] Instead, we have applied an alternative step to remove mineral residue from the HA precipitate, as more recently proposed by the IHSS:[1] the redissolved HA was passed twice over a 0.2 µm polyether sulfone membrane filter under N_2 pressure to remove the remaining minerals. The filtered HA was reprecipitated by acidifying with HCl and centrifugated. The HA pellet was subsequently suspended in UPW and dialyzed (Spectra Por 7, MWCO = 1 kDa) until the conductivity was below 5 µS cm^{-2}, and finally freeze-dried.

Proton titration

The humic acid was titrated according to the experimental and data processing protocol of Tesfa et al.[4] We followed the general strategy for model parameterization, which we adapted to the NICA-Donnan model[5] with the two-parameter ionic strength relation describing the Donnan volume dependence on ionic strength.[6] The scaled titration data are presented in Figure S1.

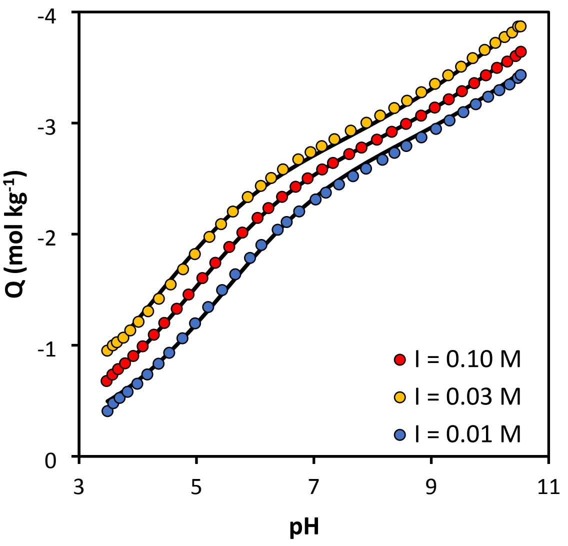

Figure S1. pH-dependent charge behavior of our HA, determined by potentiometric acid-base titration. Model lines were calculated with the optimized NICA-Donnan parameter (Table S1).

The calculations and parameterization were carried out in Matlab. In short, the procedure uses both the experimental charge (Q) versus pH data and its derivative to the pH. The derivative dQ/dpH was obtained by a cubic spline interpolation of the experimental Q vs pH curve, followed by calculating its derivative to pH.

The NICA-Donnan (ND) parameter optimization consists of two steps. In the first step, preliminary ND parameters were optimized to fit the derivative of the charge data (dQ/dpH in function of pH). This derivative is independent of the initial charge (Q_0) at the start of the titration. The initial charge (Q_0) was then calculated using these preliminary parameters. With the obtained Q_0, the absolute charge Q of our HA was calculated. A second parameter optimization was carried out using the now corrected Q versus pH and dQ/dpH versus pH data, simultaneously. The titration data and model calculations are displayed in Figure S1 and the final parameters can be found in Table S1. The Donnan volume is calculated using the two-parameter approximation:[6]

$$V_D = a\, I^b (S1)$$

Table S1. NICA-Donnan proton binding parameters for our HA (r^2=0.9996, RMSE=0.033), where $Q_{max,1}$ and $Q_{max,2}$ are the site densities of type 1 (carboxylic) and type 2 (phenolic) sites, respectively; log $\tilde{K}_{H,1}$ and log $\tilde{K}_{H,2}$ are the log median proton affinity constants of type 1 or 2 sites; m_1 and m_2 are coefficients determining the width of the proton affinity distribution, The relation between the Donnan volume and ionic strength is described by the empirical parameters a and b following Eq. S1.[6]

	$Q_{max,1}$ (mol kg^{-1})	$Q_{max,2}$ (mol kg^{-1})	log $\tilde{K}_{H,1}$	log $\tilde{K}_{H,2}$	m_1	m_2	b	a
Estimate	2.50	2.80	3.20	9.22	0.50	0.25	0.30	0.74
(±SE)	(±0.16)	(±1.06)	(±0.15)	(±0.88)	(±0.02)	(±0.07)	(±0.04)	(±0.22)

S2 Thermodynamic database

Table S2. Aqueous glyphosate speciation constants used in our surface complexation modeling. The log *K* values are for I=0 M, calculated from corresponding experimental log *K* values in 0.1 M, taken from Barja & Dos Santos Afonso,[7] using the Davies equation with D = 0.2.

**Species**	**Reaction**	**log *K***
$PMGH^{2-}$	$PMG^{3-} + H^+ \leftrightarrow PMGH^{2-}$	10.81
$PMGH_2^-$	$PMG^{3-} + 2H^+ \leftrightarrow PMGH_2^{1-}$	16.72
$PMGH_3^0$	$PMG^{3-} + 3H^+ \leftrightarrow PMGH_3^0$	19.16

Table S3. Affinity constants (log *K*) and charge distribution coefficients (Δz_i) of the surface complexes used in the S-NOM-CD model for goethite. Site densities were set to ≡FeOH = 3.45 sites nm^{-2} and $\equiv Fe_3O$ = 2.7 sites nm^{-2},[8] while the densities for ≡HNOM and $\equiv S^0$ are calculated individually as described in the main text. The capacitance values of the extended Stern layer model are $C_1 = 0.83$ F m^{-2} and $C_2 = 0.75$ F m^{-2}.[9] The ion adsorption parameters were taken from Geysels et al.[10]

Complex	$\equiv FeOH^{-0.5}$	$\equiv Fe_3O^{-0.5}$	$\equiv HNOM^{-1}$	$\equiv S^0$	Δz_0	Δz_1	Δz_2	H^+	Na^+	NO_3^-	PMG^{-3}	log *K*
$\equiv FeOH^{-1/2}$	1	0	0	0	0	0	0					0
$\equiv FeOH_2^{+1/2}$	1	0	0	0	1	0	0	1				9.3
$\equiv FeOH_2^{+1/2}$-NO_3^-	1	0	0	0	1	-1	0	1		1		8.32
$\equiv FeOH^{-1/2}$-Na^+	1	0	0	0	0	1	0		1			-0.60
$\equiv Fe_3O^{-1/2}$	0	1	0	0	0	0	0					0
$\equiv Fe_3OH^{+1/2}$	0	1	0	0	1	0	0	1				9.3
$\equiv Fe_3OH^{+1/2}$-NO_3^-	0	1	0	0	1	-1	0	1		1		8.32
$\equiv Fe_3O^{-1/2}$-Na^+	0	1	0	0	0	1	0		1			-0.60
$\equiv FePMGH^{+0.78,-0.54,-0.74}$	1	0	0	0	0.28	-0.54	-0.74	2			1	23.57
$\equiv FePMGH_2^{+0.91,+0.33,-0.74}$	1	0	0	0	0.41	0.33	-0.74	3			1	28.66
$\equiv Fe_2PMG^{+1.69,-0.95,-0.74}$	2	0	0	0	0.69	-0.95	-0.74	2			1	24.36
$\equiv Fe_2PMGH^{+1.75,-0.01,-0.74}$	2	0	0	0	0.75	-0.01	-0.74	3			1	32.60
$\equiv FeNOM^{0,-1,-0.5}$	1	0	1	0	1.5	-1.0	-0.5					0
$\equiv FeNOMH^{0,-0.5,-0}$	1	0	1	0	1.5	-0.5	0	1				2.3
$\equiv FeOH_2$-$NOM^{0.5,-1.5,-0.5}$	1	0	1	0	2	-1.5	-0.5					0.6
$\equiv S^0FeOH^{-1/2}$	1	0	0	1	0	0	0					0
$\equiv S^0FeOH_2^{+1/2}$	1	0	0	1	1	0	0	1				9.3

$\equiv S^0FeOH_2^{+1/2}\text{-}NO_3^-$	1	0	0	1	1	-1	0	1		1	8.32
$\equiv S^0FeOH^{-1/2}\text{-}Na^+$	1	0	0	1	0	1	0		1		-0.60

S3 HA-goethite composites

Table S4: HA-goethite composites prepared for the PMG adsorption experiments in systems of 20.0 g L^{-1} goethite (94 m^2 g^{-1}). The total HA level has been determined by correcting the total amount of added HA for DOC measured in the excess volume after centrifugation during the composite preparation. The HA adsorbed (%) and equilibrium HA loading Γ_{NOM} (mg HA m^{-2}) were calculated from the DOC measurement of the blank sample in each composite batch. The ratio of the equilibrium NOM loading (Γ_{NOM}) to the reference HA loading (Γ_{ref} = 1.59 mg HA m^{-2}) is used to calculate the ≡HNOM site density for each composite batch (see main text, Eq. 1).

	Initial HA level (mg HA m^{-2})	Target pH	Final pH*	Ionic strength (M)	Total HA# (mg HA m^{-2})	% HA adsorbed	Equilibrium HA loading Γ_{NOM} (mg HA m^{-2})	Γ_{NOM}/ Γ_{ref}
High	1.60	4	4.5±0.2	0.1	1.59	100	1.59	1.00
	1.60	5	5.5±0.1	0.1	1.59	97.4	1.54	0.97
	1.60	6	6.0±0.1	0.1	1.55	89.9	1.40	0.88
	1.28	7	7.0±0.1	0.1	1.26	81.7	1.03	0.65
	1.28	8	7.5±0.1	0.1	1.24	74.8	0.93	0.58
Intermediate	1.06	4	4.5±0.3	0.1	1.06	100	1.06	0.67
	1.06	5	5.5±0.3	0.1	1.06	100	1.06	0.67
	1.06	6	6.5±0.3	0.1	1.06	91.0	0.97	0.61
	1.06	7	7.5±0.1	0.1	1.05	88.1	0.93	0.58
	1.06	8	7.7±0.1	0.1	1.05	79.4	0.84	0.53
Low	0.53	4	4.4±0.3	0.1	0.53	100	0.53	0.33†
	0.53	6	6.5±0.2	0.1	0.53	100	0.53	0.33
	0.53	8	7.5±0.1	0.1	0.53	94.4	0.50	0.32
	0.53	4	4.8±0.3	0.01	0.53	100	0.53	0.33†
	0.53	6	6.5±0.3	0.01	0.53	100	0.53	0.33
	0.53	8	7.0±0.2	0.01	0.53	100	0.53	0.33

* Final pH values are given as the average of the samples, including the range of variation (±) within the samples of this composite.

# after volume correction (see main text)

† No PMG adsorption data could be reported as solution concentrations were consistently below LOD.

S4 Effect of HA on PMG adsorption

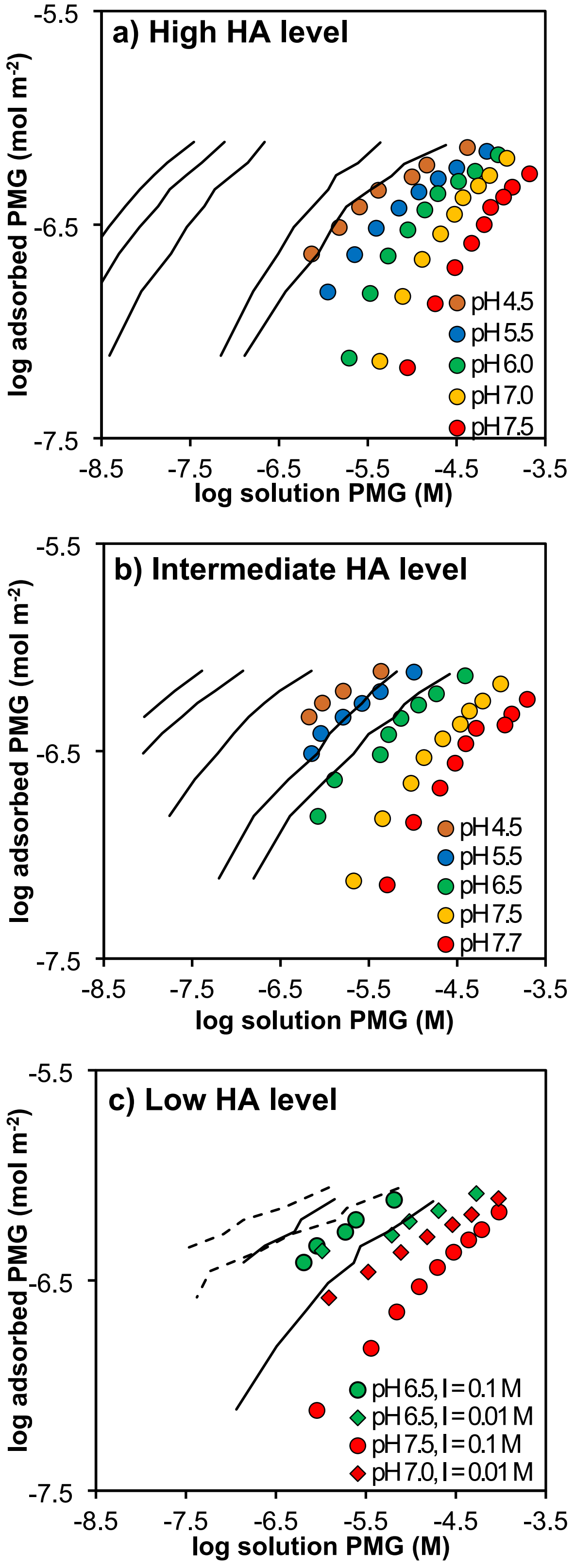

Figure S2. PMG adsorption isotherms of competitive HA-PMG systems in 10.0 g goethite L^{-1} (94 m^2 g^{-1}) in 0.1 M $NaNO_3$ (circles and solid lines) and 0.01 M $NaNO_3$ (diamonds and dashed lines), as compared to the modelled PMG adsorption in absence of HA. Model lines were calculated with the parameters from Geysels et al.[10] Total HA levels are (a) 1.6 mg HA m^{-2} for pH 4.5, 5.5, and 6.0, and 1.3 mg HA m^{-2} goethite for pH 7.0 and 7.5, (b) 1.1 mg HA $m^{-2,}$ and (c) 0.5 mg HA m^{-2}. The corresponding HA surface loadings can be found in Table S4. The pH for each isotherm provided in the legend represents the average experimental pH value, with a variation of ≤0.3 pH units. The model lines have been calculated using the individual pH values for each datapoint, resulting in a non-smooth result.

S5 Model results using basic NOM-CD

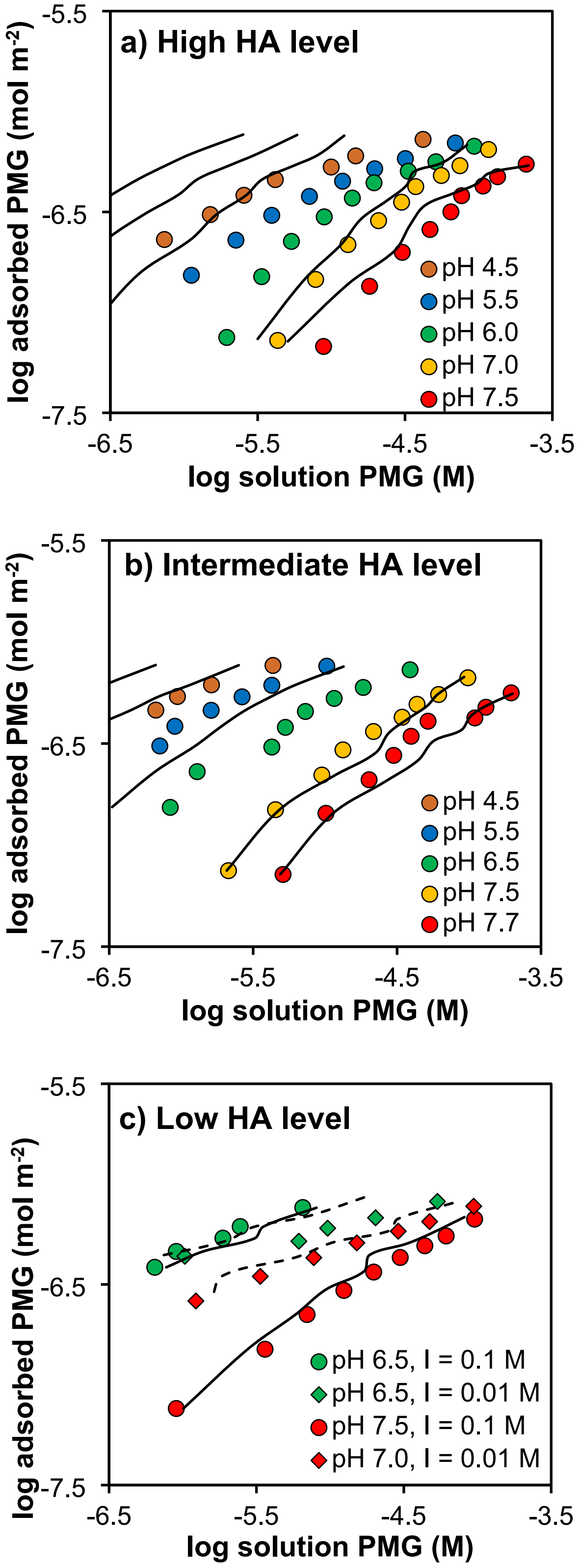

Figure S3. PMG adsorption isotherms of competitive HA-PMG systems in 10.0 g goethite L^{-1} (94 m^2 g^{-1}) in 0.1 M $NaNO_3$ (circles and solid lines) and 0.01 M $NaNO_3$ (diamonds and dashed lines). Model lines were calculated with the basic NOM-CD model (see main text) using the parameters listed in Table S3 (excluding $\equiv S^0$). We used a value of $\equiv FeNOM_T$ = 1.77 µmol m^{-2}, as derived in the main text (Figure 3). Total HA levels are (a) 1.6 mg HA m^{-2} for pH 4.5, 5.5, and 6.0, and 1.3 mg HA m^{-2} goethite for pH 7.0 and 7.5, (b) 1.1 mg HA $m^{-2,}$ and (c) 0.5 mg HA m^{-2}. The corresponding HA surface loadings can be found in Table S4. The pH for each isotherm provided in the legend represents the average experimental pH value, with a variation of ≤0.3 pH units. The model lines have been calculated using the individual pH values for each datapoint, resulting in a non-smooth result. Modeling with the basic NOM-CD model leads to overprediction of the PMG adsorption at acidic conditions.

S6 Point-by-point comparison of model results

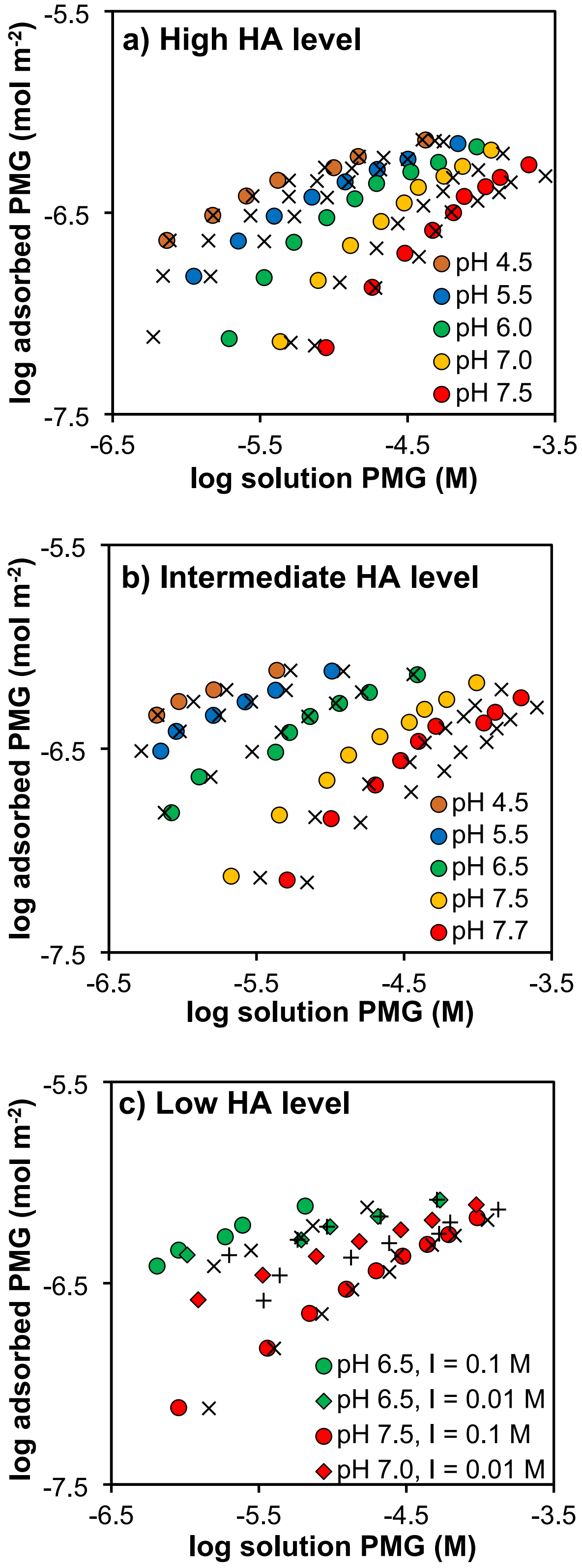

Figure S4. PMG adsorption isotherms of competitive HA-PMG systems in 10.0 g goethite L^{-1} (94 m^2 g^{-1}) in 0.1 M $NaNO_3$ (circles) and 0.01 M $NaNO_3$ (diamonds). The symbols × and + represent Steric-NOM-CD model calculations (see text) using the parameters listed in Table S3 for 0.1 M and 0.01 M ionic strength, respectively. A fixed value of $\equiv FeNOM_T$ (1.77 µmol m^{-2}) was used with $\equiv S^0_{max}$ = 2.56 ± 0.14 (0.95 CI) µmol m^{-2} being fitted as the only optimized parameter (r^2 = 0.964 and RMSE = 0.197). Total HA levels are (a) 1.6 mg HA m^{-2} for pH 4.5, 5.5, and 6.0, and 1.3 mg HA m^{-2} goethite for pH 7.0 and 7.5, (b) 1.1 mg HA $m^{-2,}$ and (c) 0.5 mg HA m^{-2}. The corresponding HA surface loadings can be found in Table S4. The pH for each isotherm provided in the legend represents the average experimental pH value, with a variation of ≤ 0.3 pH units. The modeled datapoints have been calculated using the individual pH values for each data point, resulting in a non-smooth result.

S6 Effect of ionic strength

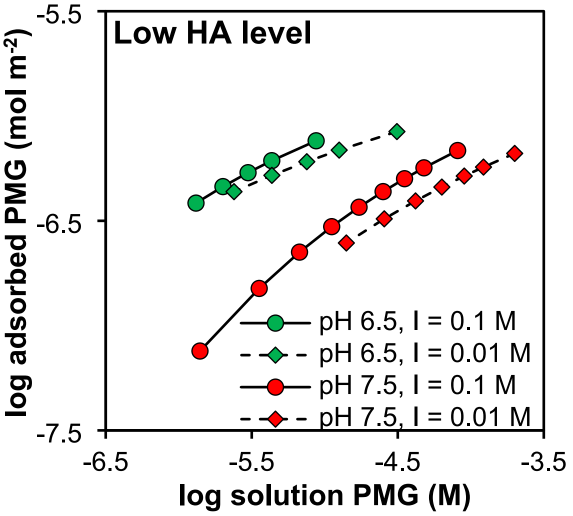

Figure S5. Modeled PMG adsorption isotherms of competitive HA-PMG systems in 10.0 g goethite L^{-1} (94 m^2 g^{-1}) in 0.1 M $NaNO_3$ (circles and solid lines) and 0.01 M $NaNO_3$ (diamonds and dashed lines) at two fixed pH levels, using Steric-NOM-CD model with parameters listed in Table S3. A fixed value of $\equiv FeNOM_T$ (1.77 μmol m^{-2}) was used with $\equiv S^0_{max}$ = 2.56. The total HA level is 0.5 mg HA m^{-2}, the corresponding HA surface loadings can be found in Table S4.

References

(1) *Isolation of IHSS Soil Fulvic and Humic Acids | IHSS*. https://humic-substances.org/isolation-of-ihss-soil-fulvic-and-humic-acids/ (accessed 2025-02-20).

(2) Swift, R. S. Organic Matter Characterization. In *SSSA Book Series*; Sparks, D. L., Page, A. L., Helmke, P. A., Loeppert, R. H., Soltanpour, P. N., Tabatabai, M. A., Johnston, C. T., Sumner, M. E., Eds.; Soil Science Society of America, American Society of Agronomy: Madison, WI, USA, 1996; pp 1011–1069. https://doi.org/10.2136/sssabookser5.3.c35.

(3) Botero, W. G.; Pineau, M.; Janot, N.; Domingos, R. F.; Mariano, J.; Rocha, L. S.; Groenenberg, J. E.; Benedetti, M. F.; Pinheiro, J. P. Isolation and Purification Treatments Change the Metal-Binding Properties of Humic Acids: Effect of HF/HCl Treatment. *Environ. Chem.* **2017**, *14* (7), 417. https://doi.org/10.1071/EN17129.

(4) Tesfa, M.; Duval, J. F. L.; Marsac, R.; Dia, A.; Pinheiro, J.-P. Absolute and Relative Positioning of Natural Organic Matter Acid–Base Potentiometric Titration Curves: Implications for the Evaluation of the Density of Charged Reactive Sites. *Environ. Sci. Technol.* **2022**, *56* (14), 10494–10503. https://doi.org/10.1021/acs.est.2c00828.

(5) Kinniburgh, D. G.; Van Riemsdijk, W. H.; Koopal, L. K.; Borkovec, M.; Benedetti, M. F.; Avena, M. J. Ion Binding to Natural Organic Matter: Competition, Heterogeneity, Stoichiometry and Thermodynamic Consistency. *Colloids Surf. Physicochem. Eng. Asp.* **1999**, *151* (1–2), 147–166. https://doi.org/10.1016/S0927-7757(98)00637-2.

(6) Koopal, L. K.; Saito, T.; Avena, M. Modified Donnan Model for Ion Binding to Small Soft Nano Particles. *Colloids Surf. Physicochem. Eng. Asp.* **2025**, *705*, 135665. https://doi.org/10.1016/j.colsurfa.2024.135665.

(7) Barja, B. C.; Dos Santos Afonso, M. An ATR−FTIR Study of Glyphosate and Its Fe(III) Complex in Aqueous Solution. *Environ. Sci. Technol.* **1998**, *32* (21), 3331–3335. https://doi.org/10.1021/es9800380.

(8) Hiemstra, T.; Riemsdijk, W. H. V.; Bolt, G. H. Multisite Proton Adsorption Modeling at the Solid/Solution Interface of (Hydr)Oxides: A New Approach. *J. Colloid Interface Sci.* **1989**, *133* (1).

(9) Weng, L.; Van Riemsdijk, W. H.; Hiemstra, T. Adsorption of Humic Acids onto Goethite: Effects of Molar Mass, pH and Ionic Strength. *J. Colloid Interface Sci.* **2007**, *314* (1), 107–118. https://doi.org/10.1016/j.jcis.2007.05.039.

(10) Geysels, B.; Hiemstra, T.; Groenenberg, J. E.; Comans, R. N. J. Glyphosate Binding and Speciation at the Water-Goethite Interface: A Surface Complexation Model Consistent with IR Spectroscopy and MO/DFT. *Water Res.* **2025**, *273*, 123031. https://doi.org/10.1016/j.watres.2024.123031.
